# Retraction Note: Diet restriction inhibits apoptosis and HMGB1 oxidation and promotes inflammatory cell recruitment during acetaminophen hepatotoxicity

**DOI:** 10.1186/s10020-020-00262-3

**Published:** 2020-12-30

**Authors:** Daniel James Antoine, Dominic P. Williams, Anja Kipar, Hugh Laverty, B. Kevin Park

**Affiliations:** 1 Medical Research Council Centre for Drug Safety Science, Department of Pharmacology and Therapeutics, Institute for Translational Medicine, University of Liverpool, Sherrington Buildings, Ashton Street, Liverpool, L69 3GE UK; 2 Department of Pharmacology and Therapeutics, Institute for Translational Medicine, University of Liverpool, Liverpool, UK; 3 Veterinary Pathology, School of Veterinary Science, University of Liverpool, Liverpool, UK

## Retraction Note to: Mol Med (2010) 16:479–490 https://doi.org/10.2119/molmed.2010.00126

The Editors-in-Chief have retracted this article (Antoine et al. 2010a) following an investigation by the University of Liverpool. The investigation concluded that data contained in this article demonstrate evidence of data manipulation and figure fabrication relating to both western blot and mass-spectrometry data and are therefore unreliable. In Fig. 2a, the blot is duplicated, cut out and flipped vertically from a blot published in Fig. 1 in the following article (Antoine et al. 2010b). In Fig. 3a, lanes in the blot have been selectively cut out and flipped horizontally between the top and bottom blot. In Fig. 5, there are inconsistencies between the stated peak masses and the x-axis. The co-authors of the article were found by the investigation not to be complicit in any research misconduct, and they have been invited to resubmit a revised version of the manuscript for further peer review. More information on the university's investigation can be found on the university website (Further update on research misconduct investigation 2020).

All authors agree to this retraction.
